# Real-Time Zero-Sequence-Voltage Estimation and Fault-Tolerant Control for an Open-Winding Five-Phase Fault-Tolerant Fractional-Slot Concentrated-Winding IPM Motor Under Inter-Turn Short-Circuit Fault

**DOI:** 10.3390/s25247655

**Published:** 2025-12-17

**Authors:** Ronghua Cui, Qingpeng Ji, Shitao Zhang, Huaxin Li

**Affiliations:** College of Electrical, Energy and Power Engineering, Yangzhou University, Yangzhou 225127, China; 15563554655@163.com (Q.J.); 19505503193@163.com (S.Z.); mz120231229@stu.yzu.edu.cn (H.L.)

**Keywords:** inter-turn short-circuit, zero-sequence voltage, feedforward compensation

## Abstract

Inter-turn short-circuit (ITSC) faults in motor drives can induce substantial circulating currents and localized thermal stress, ultimately degrading winding insulation and compromising torque stability. To enhance the operational reliability of open-winding (OW) five-phase fault-tolerant fractional-slot concentrated-winding interior permanent-magnet (FTFSCW-IPM) motor drive systems, this paper proposes a real-time fault-tolerant control strategy that provides current suppression and torque stabilization under ITSC conditions. Upon fault detection, the affected phase is actively isolated and connected to an external dissipative resistor, thereby limiting the fault-phase current and inhibiting further propagation of insulation damage. This reconfiguration allows the drive system to uniformly accommodate both open-circuit (OC) and ITSC scenarios without modification of the underlying control architecture. For OC operation, an equal-amplitude modulation scheme based on carrier-based pulse-width modulation (CPWM) is formulated to preserve the required magnetomotive-force distribution. Under ITSC conditions, a feedforward compensation mechanism is introduced to counteract the disturbance generated by the short-circuit loop. A principal contribution of this work is the derivation of a compensation term that can be estimated online using zero-sequence voltage (ZSV) together with measured phase currents, enabling accurate adaptation across varying ITSC severities. Simulation and experimental results demonstrate that the proposed method effectively suppresses fault-phase current, maintains near-sinusoidal current waveforms in the remaining healthy phases, and stabilizes torque production over a wide range of fault and load conditions.

## 1. Introduction

With the rapid advancement of transportation electrification, the reliability of traction drive systems in electric vehicles (EVs) has become a critical engineering concern [1,2]. Traction motors are required to operate continuously over wide speed ranges and elevated torque levels, often under frequent start–stop cycling. Under such demanding operating conditions, even slight insulation deterioration or localized winding defects can propagate rapidly and evolve into severe faults capable of compromising the operational safety of the entire vehicle. As a result, the design of inherently robust motor topologies, together with the development of advanced fault-detection and fault-tolerant control methodologies, has become indispensable for ensuring the dependable and safe operation of EV propulsion systems [3].

Multiphase permanent-magnet machines have emerged as a promising solution for enhancing system reliability owing to their inherent redundancy and strong fault-tolerant characteristics [4]. Compared with conventional three-phase machines, multiphase drives offer reduced torque ripple, higher power density, and the capability to maintain functional operation under partial phase failures [5,6,7]. Among them, five-phase interior permanent-magnet (IPM) machines employing fractional-slot concentrated windings (FSCW) have received significant interest in EV traction applications [8]. Their concentrated-winding structure provides low interphase magnetic coupling, improved thermal management, and strong electrical isolation between phases, effectively limiting the propagation path of fault currents and enabling improved controllability under abnormal conditions [9,10].

To further enhance system redundancy, open-winding (OW) topologies have been introduced into multiphase drive systems [11]. By using two inverters to supply the motor terminals, OW configurations provide a richer set of available voltage vectors and allow electrical decoupling between faulty and healthy phases during fault events [12,13]. In addition, OW architectures enable multi-inverter drives to share a common DC bus, making them particularly well suited for space-constrained and high–power-density EV propulsion systems.

Despite these advantages, OW five-phase FTFSCW-IPM machines remain vulnerable to inter-turn short-circuit (ITSC) faults [14]. Such faults arise from localized insulation degradation between adjacent turns, resulting in a rapidly increasing circulating current within the shorted loop, often within milliseconds [15]. This leads to intense local magnetic disturbance and heat accumulation, which induce pronounced torque pulsations. If not suppressed promptly, the fault can quickly escalate into phase-to-phase or phase-to-ground short circuits, ultimately causing catastrophic and irreversible failure [16,17]. Therefore, achieving fast estimation and effective mitigation of ITSC faults is a critical research challenge for improving the reliability of modern electric drive systems.

Recent advances in ITSC diagnosis techniques [18,19,20,21,22,23,24,25,26,27,28,29] have led to a diverse set of approaches that can be broadly categorized into model-based, signal-analysis-based, high-frequency–injection-based, and data-driven methodologies. Model-based schemes rely on analytical representations of faulty machines and infer ITSC indicators from parameter or state deviations; examples include hybrid flux estimators that capture compensation-voltage perturbations but are sensitive to parameter mismatches [20], Luenberger and extended-state observers that extract severity information from voltage residuals [21], and sliding-mode-observer designs with SOGI-based EMF reconstruction that enhance noise immunity while simultaneously detecting, locating, and quantifying fault severity [22].

Signal-analysis-based techniques extract characteristic patterns from measured current, voltage, or flux, such as complex-signal analysis for induction machines [23], magnetic-symmetry indices for evaluating spatial field distortion under ITSC conditions [24], and phase-estimation-based demodulation that remains effective under nonstationary speed operation [25]. Voltage-demodulation methods have also been applied to multiphase machines, where second-harmonic distortion in d-q axis voltages provides a quantitative indicator of fault severity [26].

High-frequency signal-injection strategies further improve diagnostic sensitivity at low speeds or standstill by exploiting ITSC-induced asymmetry; for instance, D-axis high-frequency impedance extraction enables reliable detection in traction motors [27], albeit at the cost of additional injection hardware or careful compensation of inverter nonlinearity.

More recently, data-driven and machine-learning frameworks have demonstrated strong performance in noisy, nonlinear, and multi-fault scenarios. Robust fault-classification models capable of jointly identifying ITSC and demagnetization conditions have been proposed [28], and deep-learning schemes such as Siamese convolutional neural networks achieve high diagnostic accuracy under limited fault samples by leveraging metric-learning-based feature extraction [29]. Although these methods offer excellent accuracy and robustness, they generally require extensive labeled datasets and may suffer from limited interpretability, constraining their applicability in safety-critical traction systems.

Beyond detection and severity estimation, several studies have begun exploring fault-tolerant control strategies specifically tailored to ITSC scenarios. For instance, concentric-winding 3-3-phase permanent-magnet drives have been designed to inherently reduce fault-current circulation and retain post-fault torque capability [30]. Current-injection-based FTC has also been proposed for PMSMs, where the injected sequence components suppress ITSC-induced torque ripple while maintaining acceptable torque output [31]. More recently, disturbance-observer-based methods have enabled unified diagnosis and FTC operation in five-phase PMSMs, directly extracting ITSC disturbance signatures for real-time compensation [32]. These emerging strategies highlight a growing interest in bridging the gap between ITSC diagnostics and actionable real-time control. In addition to these methods, previous work has shown that torque ripple caused by ITSC faults can also be mitigated through feedforward compensation in OW five-phase FTFSCW-IPM drives, where the short-circuit disturbance is estimated in real time using measured phase voltage information [33]. Although this approach effectively suppresses torque pulsation, it depends on an additional voltage sensor connected to the isolated phase, which increases hardware complexity and restricts deployment in space-constrained traction applications.

In this study, an external dissipative resistor is still introduced to limit the fault current and avoid thermal expansion of the short-circuited turns. However, unlike voltage-sensor-dependent methods, the proposed strategy does not require measuring the phase voltage across the disconnected winding. Instead, the disturbance associated with the short-circuit loop is inferred from the zero-sequence voltage (ZSV) and the measured phase currents of the healthy windings, which are already available in standard drive hardware. As a result, the proposed method enables real-time compensation without modifying the existing sensing architecture or current controller structure.

The present work addresses these challenges by proposing a real-time estimation and fault-tolerant control strategy tailored to OW five-phase FTFSCW-IPM drives operating under ITSC faults. Specifically, the method exploits the intrinsic topological characteristics of the OW configuration to reconstruct the ITSC disturbance through zero-sequence voltage (ZSV) information. Unlike conventional ITSC mitigation strategies that require structural modification of the controller or explicit short-circuit-loop sensing, the proposed approach operates can be seamlessly incorporated into existing vector-control frameworks. By actively opening the faulted phase and introducing an external dissipative resistor, the short-circuit-loop energy is effectively limited, preventing further fault escalation while maintaining safe operating conditions. The main contributions of this paper can be summarized as follows:

(1) A ZSV-based real-time estimation scheme is developed to extract the ITSC-induced disturbance term without requiring additional sensors.
(2) A fault-tolerant control strategy is formulated that compensates the disturbance using feedforward terms while preserving the original controller structure.
(3) Robust fault mitigation is demonstrated under varying short-circuit turn ratios and dynamic load conditions, verifying the practical applicability of the method.

The remainder of this paper is organized as follows. Section 2 introduces the OW five-phase FTFSCW-IPM drive system. Section 3 presents the proposed fault-tolerant control strategy with real-time estimation of ZSV. Section 4 provides simulation and experimental validation, and Section 5 concludes the paper.

## 2. Architecture and Characteristics of the OW Five-Phase FTFSCW-IPM Drive System

**Figure 1:**
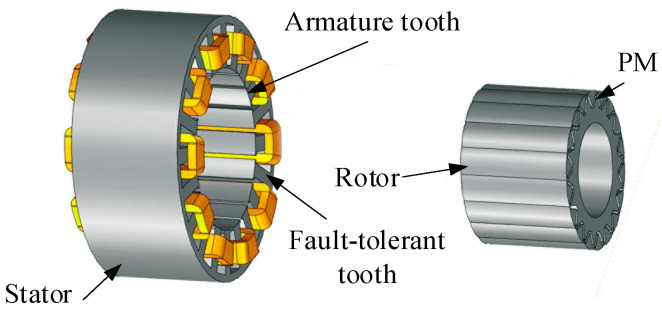
**3D** view of the five-phase FTFSCW-IPM motor.

**Figure 2:**
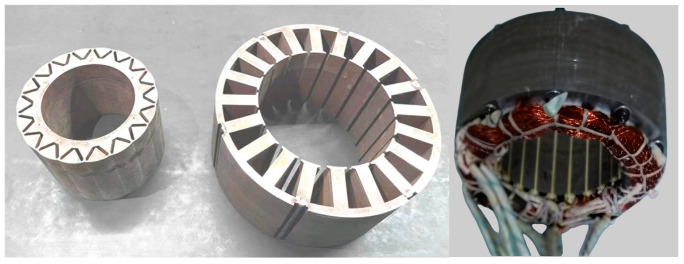
Prototype of the five-phase FTFSCW-IPM motor.

The motor investigated in this study is a five-phase fault-tolerant fractional-slot concentrated-winding interior permanent-magnet (FTFSCW-IPM) machine (self-developed prototype, Yangzhou University, Yangzhou, China). The three-dimensional model and the prototype of this motor are shown in Figure 1 and Figure 2, respectively.

**Figure 3:**
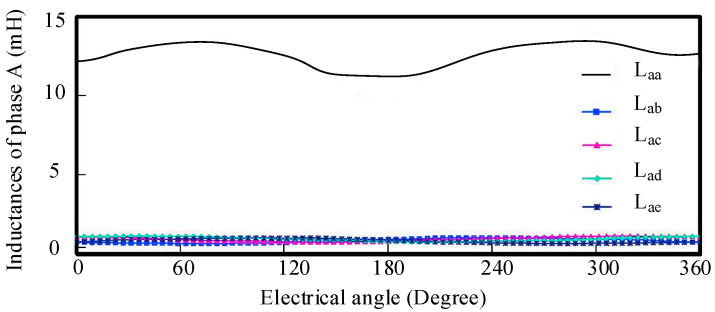
Comparison of self-inductance and mutual inductance of phase A winding.

As illustrated in these figures, the machine adopts a fault-tolerant tooth structure that provides effective electrical, magnetic, thermal, and physical isolation between phase windings, both under healthy and faulty operating conditions. The measured inductances of the motor are presented in Figure 3. The results show that the self-inductance of each phase is 13.5 mH, while the mutual inductance is only 0.035 mH.

Thus, the mutual inductance corresponds to merely 0.259% of the self-inductance, confirming that the interphase coupling is inherently extremely weak for this FTFSCW-IPM topology. Consequently, it is reasonable to neglect the mutual inductances in the modeling process. Furthermore, the relatively large self-inductance helps effectively limit the short-circuit current during inter-turn short-circuit (ITSC) conditions.

**Figure 4:**
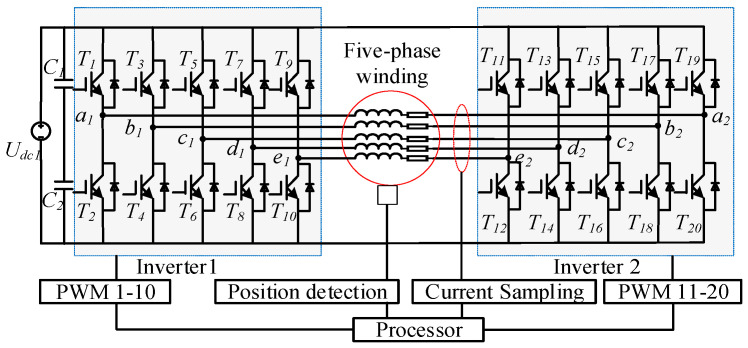
Electrical topology of the OW five-phase FTFSCW-IPM motor.

The architecture of the OW five-phase FTFSCW-IPM drive employed in this study is shown in Figure 4. The system consists of a common DC bus supplying two conventional two-level inverters, each connected to one end of the five-phase stator winding. This dual-inverter configuration effectively reduces electrical coupling between phases and provides a flexible platform for implementing fault-tolerant control, as the two bridges can independently regulate phase voltages during both healthy and post-fault operation.

**Figure 5:**
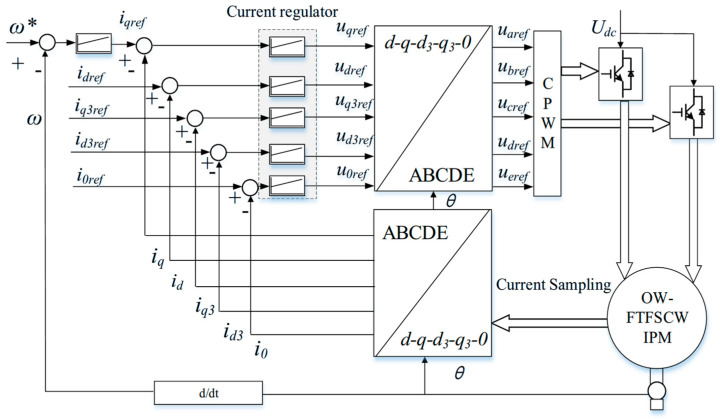
Control architecture of the proposed OW drive system under healthy operation.

The control scheme is depicted in Figure 5. During healthy operation, the measured five-phase currents are transformed into the d–q–d_3_–q_3_–0 subspaces, where independent proportional–integral (PI) controllers regulate the current components. Their outputs are mapped back to the stationary frame through the inverse coordinate transformation, generating the reference phase voltages. These voltages are then synthesized using carrier-based pulse-width modulation (CPWM), which is preferred over space-vector PWM in OW five-phase drives because SVPWM requires handling up to 211 switching vectors, leading to significantly higher computational complexity.

Due to the characteristics of fractional-slot concentrated windings, the five-phase stator produces inherent third-harmonic and zero-sequence subspaces. If not properly regulated, these components can introduce additional copper losses, distort the air-gap flux, and cause torque ripple. Therefore, during normal operation, the corresponding harmonic currents are forced to zero through closed-loop regulation, ensuring that only the fundamental d–q currents contribute to torque production. This improves drive efficiency, reduces electromagnetic vibration, and enhances overall dynamic performance.

## 3. Fault-Tolerant Control Strategy with ZSV-Based Real-Time Estimation

When an ITSC fault occurs, one of the first steps toward preventing fault escalation is to isolate the affected phase. After this isolation, the drive effectively operates with four healthy phases, and a fault-tolerant control (FTC) strategy must be deployed to keep the torque within acceptable bounds. To accommodate both the open-circuit (OC) fault and ITSC scenarios, the proposed framework uses a unified reconstruction procedure: the faulted phase is disconnected from the inverter and routed through an external dissipative resistor, such that there is only a small residual current flow in the healthy portion of the winding, while the remaining phases are modulated to preserve smooth torque production in the OW five-phase FTFSCW-IPM drive.

### 3.1. Current Reallocation FTC Strategy for Active Open-Circuit Faults

When phase A becomes open-circuited, the other four phases must collectively generate the required magnetomotive force (MMF). Since the spatial MMF generated by the five-phase set must remain unchanged to avoid torque dip, the control objective redistributes the current among the four functional phases so that the fundamental MMF vector maintains its amplitude and orientation.

Let $i_{x}^{\prime }$ (*x* = *B*, *C*, *D*, *E*) denote the post-fault phase currents. The fundamental MMF produced by the four phases can be expressed as

(1) $$\left\{\begin{array}{ll} 2.5I_{m}\cos\theta = & {i^{\prime }}_{B}\cos72{}^{\circ}+{i^{\prime }}_{C}\cos144{}^{\circ}+{i^{\prime }}_{D}\cos\;(-144{}^{\circ})\\ & +{i^{\prime }}_{E}\cos\;(-72{}^{\circ})\\ 2.5I_{m}\sin\theta = & {i^{\prime }}_{B}\sin72{}^{\circ}+{i^{\prime }}_{C}\sin144{}^{\circ}+{i^{\prime }}_{D}\sin\;(-144{}^{\circ})\\ & +{i^{\prime }}_{E}\sin\;(-72{}^{\circ}) \end{array}\right.$$

where *I_m_* is the desired equivalent MMF amplitude and θ is the rotor electrical position. To balance the current stress among the four healthy phases, the equal-amplitude current allocation principle is adopted.

(2) $${i^{\prime }}_{B}=-{i^{\prime }}_{D}\;\;\;{i^{\prime }}_{C}=-{i^{\prime }}_{E}$$

Substituting (2) into (1) yields the required magnitude of the post-fault synthetic current:

(3) $$\left\{\begin{array}{l} {i^{\prime }}_{B}=1.382I_{m}\cos(\theta -36{}^{\circ})\\ {i^{\prime }}_{C}=1.382I_{m}\cos(\theta -144{}^{\circ})\\ {i^{\prime }}_{D}=1.382I_{m}\cos(\theta +144{}^{\circ})\\ {i^{\prime }}_{E}=1.382I_{m}\cos(\theta +36{}^{\circ}) \end{array}\right.$$

Thus, compared with healthy operation, the four remaining phases must increase their current by approximately 38.2% to compensate for the missing phase.

**Figure 6:**
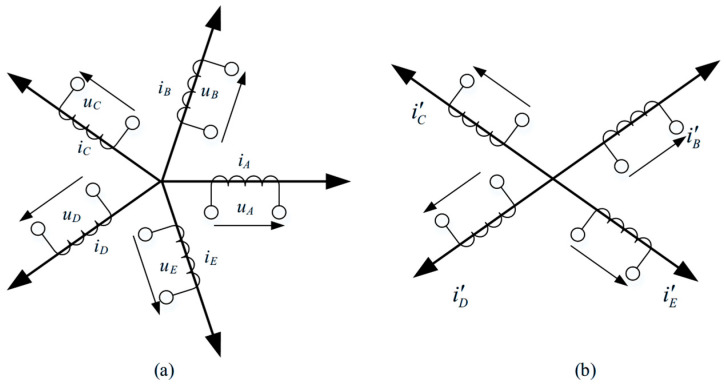
Vector diagram of phase currents under (**a**) normal operation and (**b**) an open-phase condition.

The current trajectories before and after the OC fault are illustrated in Figure 6a,b. As seen from the figure, enforcing equal-magnitude currents effectively suppresses MMF distortion during the transition, ensuring smooth torque. However, direct hysteresis control tends to produce irregular switching and elevated losses. To address this, a CPWM strategy is adopted for the OC state, enabling accurate phase-voltage synthesis while maintaining current-control bandwidth.

Because torque production occurs exclusively in the α–β plane, the transformed coordinate system must be redefined after phase A is removed. The modified Clarke transformation for the four-phase case is constructed by enforcing orthogonality among the α, β, γ, and zero-sequence subspaces. The corresponding inverse Clarke matrix is expressed as:

(4) $$T_{F}^{-1}=\left[\begin{array}{cccc} 1.118 & 0.8123 & 1.382 & 1.382\\ -1.118 & 0.8123 & -1.382 & 1.382\\ -1.118 & -0.8123 & 1.382 & 1.382\\ 1.118 & -0.8123 & -1.382 & 1.382 \end{array}\right]$$

The associated Park matrix follows directly from the α–β definition:

(5) $$T_{P}=\left[\begin{array}{cccc} \cos\theta & \sin\theta & 0 & 0\\ -\sin\theta & \cos\theta & 0 & 0\\ 0 & 0 & 1 & 0\\ 0 & 0 & 0 & 1 \end{array}\right]$$

These transformations allow the OW five-phase drive to maintain nominal torque-generation ability during OC operation, provided that the reconstructed phase currents satisfy the equal-amplitude requirement in (3).

### 3.2. Unified FTC Strategy for Combined OC and ITSC Faults via ZSV-Based Disturbance Estimation

**Figure 7:**
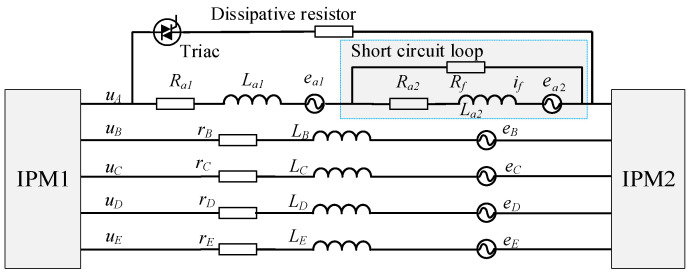
Equivalent circuit representation of the OW FTFSCW-IPM motor under an ITSC fault.

Building on the OC-fault compensation framework discussed previously, this subsection extends the strategy to conditions where an OC fault and an ITSC fault coexist in the same phase. The electrical configuration of the OW five-phase FTFSCW-IPM motor under ITSC conditions is shown in Figure 7, where the affected phase is isolated from the inverter bridge and interfaced with an external dissipative resistor.

When an ITSC fault is detected, the Triac can be triggered, and resistor *R* is inserted in series with the damaged phase, which serves to suppress the current stress within the partially shorted winding and simultaneously enables real-time acquisition of the phase-voltage information needed for ZSV-based disturbance estimation. This measurement capability is essential because the circulating current *i_f_* within the short-turn loop cannot be directly sensed.

The severity of insulation degradation is characterized by the short-circuit resistance *R_f_*. The measurable current $i_{A}^{\prime }$ represents the portion of the phase-A current flowing through the healthy turns, while *i_f_* corresponds to the short-circuit loop current. Parameters (*R_a_*_1_, *L_a_*_1_) denote the electrical characteristics of the healthy segment of phase A, whereas (*R_a_*_2_, *L_a_*_2_) represent those of the short-circuited turns. The five self-inductances (*L_x_*, *x* = *A*–*E*) are assumed equal to *L_s_*, and the mutual inductances *M_xy_* (*x* ≠ *y*) are approximated as *L_m_*. The back-EMFs of the individual phases are denoted by *e_x_* (*x* = *A*–*E*).

Under a pure OC condition, the disturbance induced by removing phase A can be fully compensated through the Clarke-Park transformations in (4) and (5), without altering the controller architecture. However, when the OC fault is accompanied by an ITSC fault, the back-EMF of phase A continues to energize the short-circuited turns through the external resistor, even though the phase is electrically open to the inverter. This behavior means that torque production is influenced not only by the currents of the remaining four phases but also by the electromotive action of the internal short-circuit loop. Under these conditions, the torque is expressed by

(6) $$\begin{array}{ll} T_{ef} & =(e_{A}{i^{\prime }}_{A}+e_{B}{i^{\prime }}_{B}+e_{C}{i^{\prime }}_{C}+e_{D}{i^{\prime }}_{D}+e_{E}{i^{\prime }}_{E}-e_{f}i_{f})/\omega_{m}\\ & =[e_{A}({i^{\prime }}_{A}-\mu i_{f})+e_{B}{i^{\prime }}_{B}+e_{C}{i^{\prime }}_{C}+e_{D}{i^{\prime }}_{D}+e_{E}{i^{\prime }}_{E}]/\omega_{m} \end{array}$$

where *ω_m_* is the mechanical angular velocity, *μ* is the short-circuit turn ratio, *e_f_* denotes the back-EMF induced in the shorted turns, and $i_{A}^{\prime }$ is the measurable current through resistor *R*. The disturbance term *e_A_*(*μi_f_* − $i_{A}^{\prime }$) directly perturbs torque production by introducing a pulsating MMF component, thereby degrading the steady-state performance of the drive.

**Figure 8:**
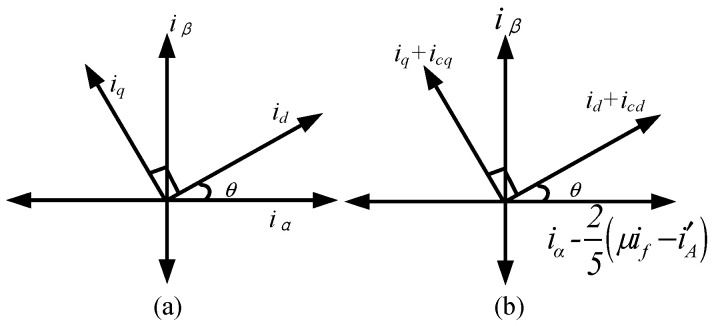
Current components in the stationary and synchronous reference frames. (**a**) OC-fault case. (**b**) ITSC-affected phase connected to the dissipative branch.

The current behavior under these conditions is illustrated in Figure 8. When only an OC fault is present, the current vectors in both stationary and synchronous frames follow the relationships defined in (8), as shown in Figure 8a. When an ITSC fault is also present, the measurable current $i_{A}^{\prime }$ generates an additional projection onto the α-axis, resulting in the modified stationary-frame currents shown in Figure 8b. This modification effectively adds a disturbance term of 0.4(*μi_f_* − $i_{A}^{\prime }$) to the α-component.

Let *i_cd_* and *i_cq_* denote the compensation currents injected into the d-q axis of the synchronous frame. The transformation between stationary and synchronous components can then be written as

(7) $$\left(i_{d}+i_{cd}\right)\cos\theta -\left(i_{q}+i_{cq}\right)\sin\theta =i_{\alpha }-\frac{2}{5}(\mu i_{f}-{i^{\prime }}_{A})$$

(8) $$\left(i_{d}+i_{cd}\right)\sin\theta +\left(i_{q}+i_{cq}\right)\cos\theta =i_{\beta }$$

From which the required compensation currents are obtained as

(9) $$\left\{\begin{array}{l} i_{cd}=-\frac{2}{5}(\mu i_{f}-{i^{\prime }}_{A})\cos\theta \\ i_{cq}=\frac{2}{5}(\mu i_{f}-{i^{\prime }}_{A})\sin\theta \end{array}\right.$$

These expressions show that the compensation depends on four quantities: the short-circuit turn ratio *μ*, the short-circuit loop current *i_f_*, the measurable phase-A current $i_{A}^{\prime }$, and the rotor electrical position *θ*. Under normal operation, a standard d–q double closed-loop current controller is employed. To preserve the existing control architecture while addressing both OC and ITSC faults, real-time availability of the disturbance term (*μi_f_* − $i_{A}^{\prime }$) is essential.

Because the short-circuit loop current *i_f_* cannot be directly measured when phase A is open-circuited, the analysis relies on the stator-voltage equations under ITSC conditions. With mutual inductances neglected, these equations take the form

(10) $$u_{A}=R_{s}{i^{\prime }}_{A}-\mu R_{s}i_{f}+L_{s}p{i^{\prime }}_{A}-\mu L_{s}p{i^{\prime }}_{A}+e_{A}$$

(11) $$u_{B}=R_{s}{i^{\prime }}_{B}+L_{s}p{i^{\prime }}_{B}+e_{B}$$

(12) $$u_{C}=R_{s}{i^{\prime }}_{C}+L_{s}p{i^{\prime }}_{C}+e_{C}$$

(13) $$u_{D}=R_{s}{i^{\prime }}_{D}+L_{s}p{i^{\prime }}_{D}+e_{D}$$

(14) $$u_{E}=R_{s}{i^{\prime }}_{E}+L_{s}p{i^{\prime }}_{E}+e_{E}$$

(15) $$i_{0}=({i^{\prime }}_{A}+{i^{\prime }}_{B}+{i^{\prime }}_{C}+{i^{\prime }}_{D}+{i^{\prime }}_{E})/5$$

(16) $$u_{0}=(u_{A}+u_{B}+u_{C}+u_{D}+u_{E})/5$$

where *p* denotes the Laplace operator, and *u*_0_ and *i*_0_ represent the ZSV and zero-sequence current, respectively. When phase A is open-circuited and connected to the external resistor, summing the voltage equations in (10)–(16) gives

(17) $$5R_{s}i_{0}+5L_{s}pi_{0}-\mu R_{s}i_{f}-\mu L_{s}pi_{f}=5u_{0}$$

Applying the Laplace transform yields

(18) $$\mu i_{f}-{i^{\prime }}_{A}=i_{0F}-\frac{5u_{0}}{L_{s}s+R_{s}}$$

(19) $$i_{0F}={i^{\prime }}_{B}+{i^{\prime }}_{C}+{i^{\prime }}_{D}+{i^{\prime }}_{E}$$

**Figure 9:**
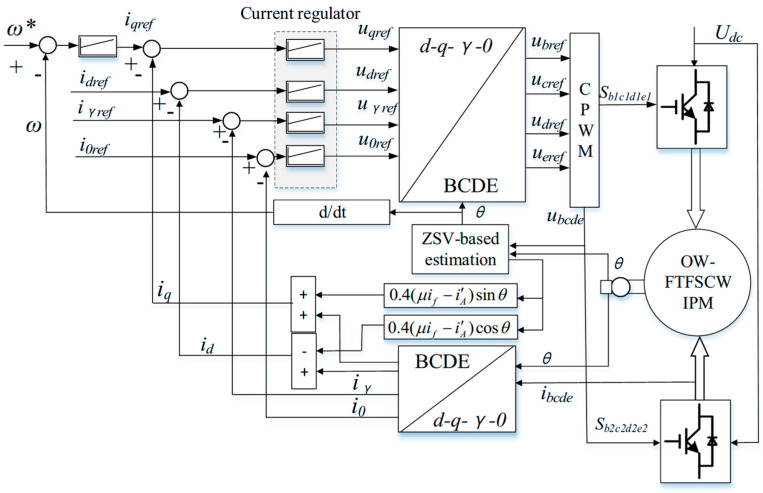
Fault-tolerant control structure incorporating ZSV-based compensation for ITSC operation.

As indicated by (18), the term ‘*μi_f_* − $i_{A}^{\prime }$’ can be estimated in real time from the ZSV and the currents of the four healthy phases, even when phase A is open-circuited. From (16), the ZSV is obtained as the sum of the individual phase voltages. For phase A, the voltage *u_A_* can be readily computed using Ohm’s law, since the current $i_{A}^{\prime }$ flowing through the external resistor R is directly measurable. The voltages of the remaining phases *u_x_* (*x* = *B*, *C*, *D*, *E*) can be calculated from their corresponding inverter duty cycles. Consequently, even when the short-circuit turn ratio μ and the short-circuit current *i_f_* vary dynamically during fault-tolerant operation, (18) and (19) provides an accurate and sensorless estimation of the disturbance term required for compensation. Once (*μi_f_* − $i_{A}^{\prime }$) is estimated, the reference voltage vector for the four healthy phases is computed as

(20) $$\begin{array}{l} \left[\begin{array}{c} u_{B}^{*}\\ u_{C}^{*}\\ u_{D}^{*}\\ u_{E}^{*} \end{array}\right]=T_{F}^{-1}T_{P}^{-1}\left[\begin{array}{c} \left(k_{dp}+\frac{k_{di}}{s})\times [i_{d}^{*}-i_{d}+0.4(\mu i_{f}-{i^{\prime }}_{A})\cos\theta \right]\\ \left(k_{qp}+\frac{k_{qi}}{s})\times [i_{q}^{*}-i_{q}-0.4(\mu i_{f}-{i^{\prime }}_{A})\sin\theta \right]\\ k_{\gamma p}+\frac{k_{\gamma i}}{s}\\ k_{op}+\frac{k_{0i}}{s} \end{array}\right]+\left[\begin{array}{c} e_{B}\\ e_{C}\\ e_{D}\\ e_{E} \end{array}\right] \end{array}$$

where *k_xp_* and *k_xi_* are the proportional and integral gains associated with the d–q–γ–0 subspaces. For the sake of brevity, the detailed derivation of (20) is provided in Appendix A. The complete control structure under combined OC and ITSC faults is depicted in Figure 9.

As shown in Figure 9, the compensation term is injected into the d–q current regulators, while harmonic-plane regulation is performed on *i_γ_*, replacing the conventional d_3_–q_3_ regulation used in fully healthy conditions. Importantly, the underlying control architecture, including parameter settings, PI regulators, and PWM generation, remains unchanged. This allows the proposed method to be implemented with minimal modification to existing drive systems while achieving significant suppression of torque ripple during ITSC faults.

## 4. Discussion

### 4.1. Simulation Results

#### 4.1.1. Simulation Validation

To assess the performance of the proposed fault-tolerant control strategy under ITSC conditions, a MATLAB R2023b/Simulink model was constructed according to the system configuration illustrated in Figure 9. The motor was simulated at 600 r/min under a 6 N·m load torque. For the reconstructed topology, an external resistor of 150 Ω was selected so that the current in the short-circuited phase remained below 1 A. To evaluate the torque performance under different operating conditions, the torque ripple is defined as the ratio of the peak-to-peak torque to the average torque.

**Figure 10:**
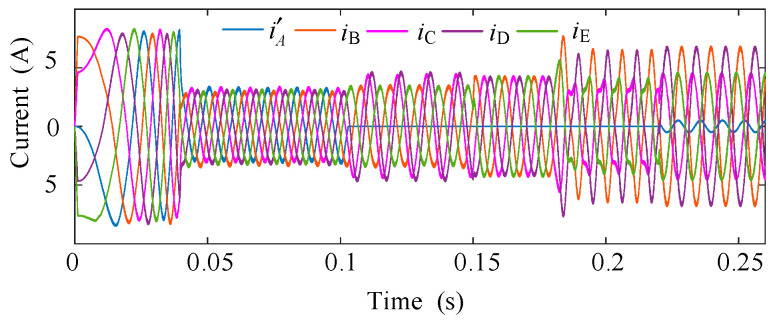
Five-phase current waveforms during the transition to fault-tolerant operation.

**Figure 11:**
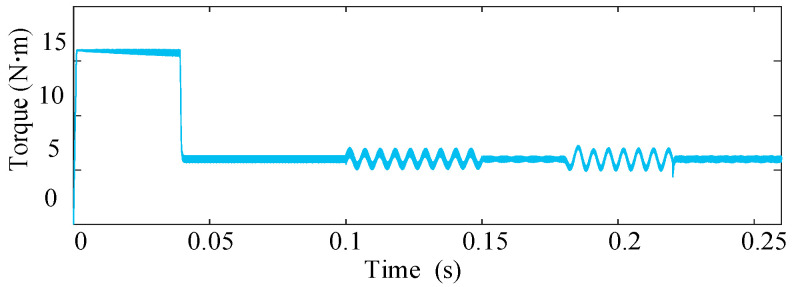
Torque response during the transition to fault-tolerant operation.

Figure 10 and Figure 11 depict the simulated phase currents and torque during the entire ITSC fault process for *μ* = 0.577 and *R_f_* = 2 Ω. At *t *= 0.1 s, an OC fault is introduced in phase A. This causes an immediate disturbance in the system, resulting in noticeable phase current distortions and a pronounced torque ripple of 24%. At *t *= 0.15 s, the open-circuit FTC is activated, and the system compensates for the imbalance by modulating the currents of the remaining four healthy phases. Although the system begins to recover, some imbalance in the current still persists during the early stages of the FTC operation. When the system reaches *t *= 0.18 s, an inter-turn short-circuit (ITSC) fault is introduced in phase A, further deteriorating the system performance. The phase current distortion becomes more pronounced, and the torque ripple increases again, as evident in the figures. The torque output exhibits a significant disturbance, with the torque ripple rising to 28% relative to the average torque, reflecting the added complexity of the ITSC fault. Finally, at *t* = 0.22 s, the proposed ITSC fault-tolerant control based on zero-sequence voltage (ZSV) estimation is activated. This compensation strategy directly estimates the disturbance from the ZSV and remaining phase currents. Beyond this point, the torque oscillations are effectively attenuated. The torque ripple is maintained at a low level of 2.8%, and the phase currents recover their sinusoidal shapes.

Furthermore, the current $i_{A}^{\prime }$ flowing through the healthy portion of phase A remains small owing to the dissipative resistor, thereby reducing the likelihood of further insulation deterioration.

**Figure 12:**
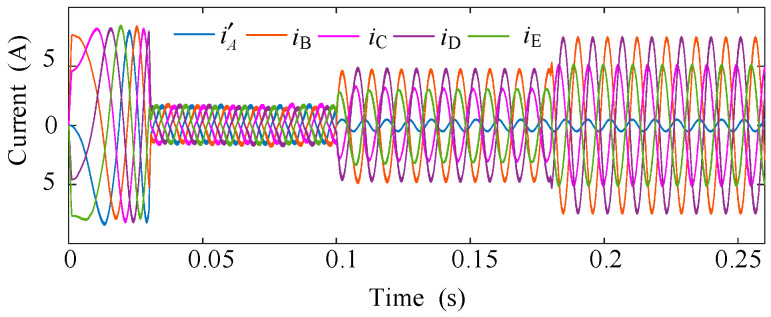
Phase-current waveforms under varying load conditions.

**Figure 13:**
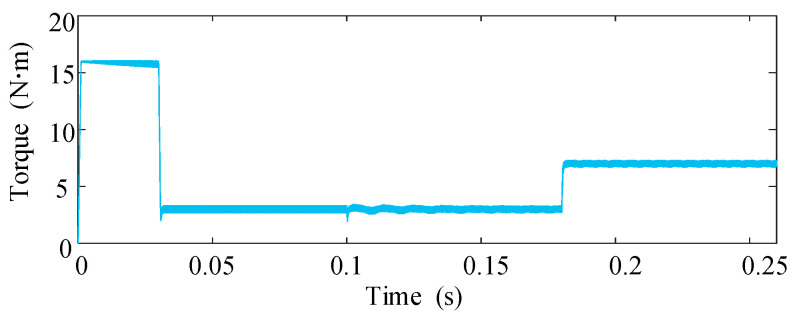
Torque response under varying load conditions.

The dynamic performance of the proposed scheme is further evaluated in Figure 12 and Figure 13, where the drive operates under varying load torque. At *t *= 0.1 s, an ITSC fault occurs and the fault-tolerant control is activated without delay. As shown, the torque maintains a stable profile during the transition, indicating that the disturbance induced by the circulating short-circuit current is effectively compensated. When the load torque is increased from 3 N·m to 7 N·m at *t *= 0.18 s, the phase-current amplitudes rise accordingly while preserving good sinusoidal quality, and the torque response exhibits no overshoot or ripple amplification. These observations confirm that the proposed method retains strong disturbance-rejection capability under dynamic loading.

**Figure 14:**
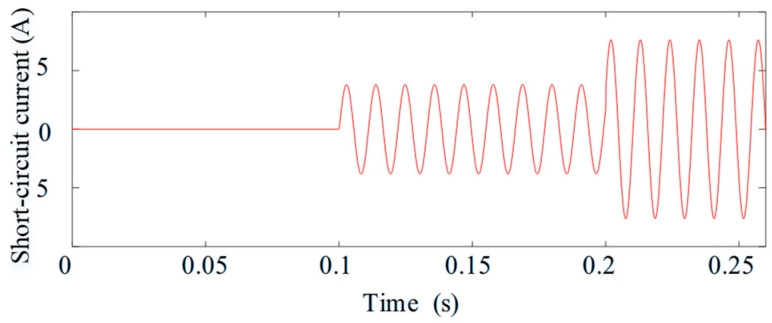
Short-circuit current waveforms under varying short-circuit turn ratios.

**Figure 15:**
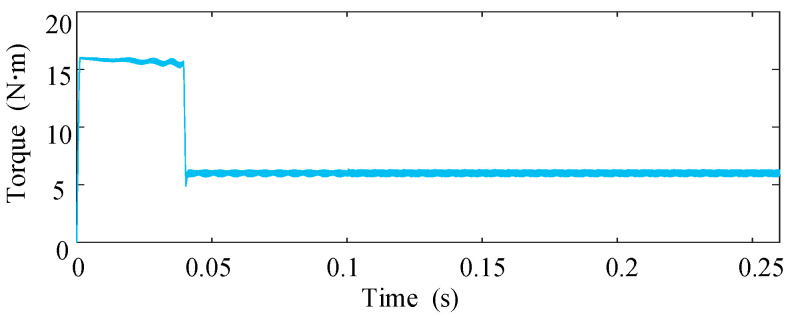
Torque response under varying short-circuit turn ratios.

Figure 14 and Figure 15 investigate the behavior of the system when the short-circuit turn ratio varies during fault-tolerant operation. The ITSC fault occurs at *t* = 0.1 s. After *t* = 0.2 s, μ gradually increases from 0.18 to 0.577, emulating a progressive deterioration of the fault. As expected, the short circuit current amplitudes increase with higher ITSC severity; however, the torque remains stable and free of oscillations throughout the transition. This result demonstrates that the control reconstruction and compensation strategy remain effective despite changes in the internal fault severity.

**Figure 16:**
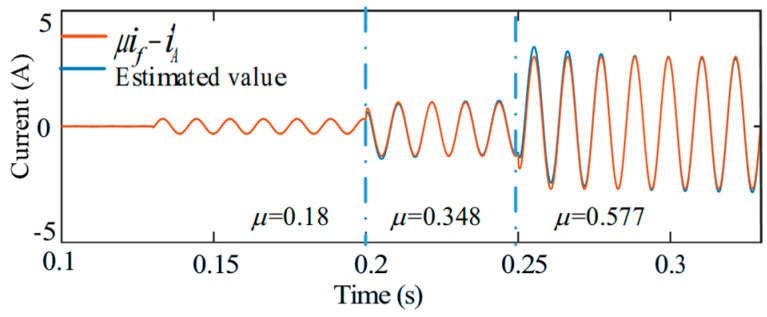
Estimated feedforward compensation under varying short-circuit turn ratios.

Finally, Figure 16 evaluates the real-time estimation accuracy of the compensation term derived from (18) and (19).

It can be seen from Figure 16 that the estimated disturbance closely tracks its actual value during the entire variation of *μ*, confirming that the proposed ZSV-based estimator provides reliable and accurate compensation information even when the fault level evolves dynamically.

#### 4.1.2. Robustness Analysis

To further evaluate the robustness of the proposed ZSV estimation and the corresponding FTC scheme under non-ideal conditions, comprehensive simulations were carried out by considering parameter variations, magnetic saturation, inverter nonlinearities, and measurement noise. In all robustness tests, an ITSC fault was intentionally introduced at *t* = 0.15 s, and the proposed FTC strategy was activated simultaneously. The robustness of the ZSV estimation was first analyzed at the signal level, followed by the assessment of its final impact on the torque performance.

It should be noted that, in the control model, the zero-sequence voltage (ZSV) is originally obtained from the inverter switching states in the form of a PWM duty-cycle-based pulse signal. To obtain a physically meaningful continuous ZSV signal for feedforward compensation, the raw switching signal is processed through an equivalent first-order inertial link *U*_0_/(*sL_s_* + *R_s_*), which represents the electrical dynamics of the stator winding. This filtered ZSV signal is exactly the same as that used in the proposed compensation strategy throughout the paper. Therefore, all the robustness analyses presented in this section are performed on the filtered continuous ZSV signal after the inertial link.

1. Influence of Stator Resistance R_s_ Variations

**Figure 17:**
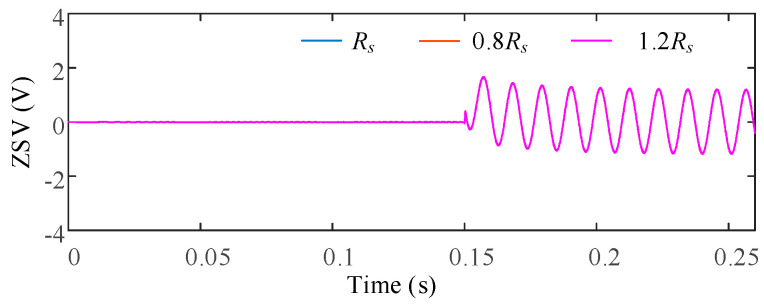
ZSV under different *R_s_*.

**Figure 18:**
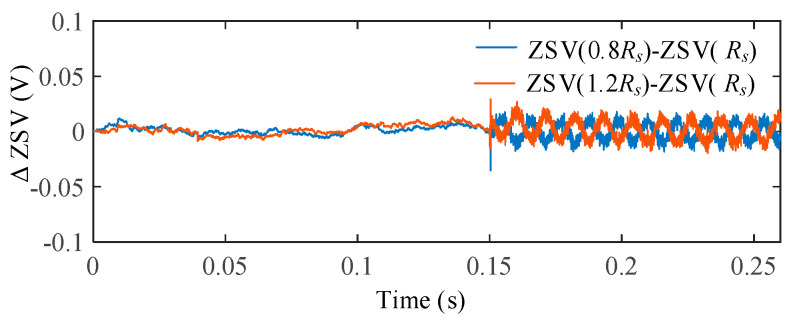
ΔZSV with respect to the nominal *R_s_* under stator resistance variations.

To evaluate the influence of stator resistance uncertainty, the stator resistance was varied as 0.8*R_s_*, 1.0*R_s_*, and 1.2*R_s_*, representing typical temperature-induced parameter deviations. Figure 17 shows the ZSV obtained under different resistance conditions after the ITSC fault occurs at *t* = 0.15 s. For a clearer comparison, Figure 18 illustrates the deviation of the ZSV signals under parameter variations from the nominal case.

It is observed that the ZSV waveforms under different resistance conditions almost overlap, indicating a very weak sensitivity of the ZSV estimation to *R_s_* deviations. Quantitatively, the ZSV deviation caused by resistance variation is measured to be only 1.15%, confirming that the proposed ZSV estimation is highly robust against *R_s_* uncertainty.

2. Influence of Inductance *L_s_* Variations and Magnetic Saturation

The influence of phase inductance uncertainty was investigated by varying the inductance as 0.8*L_s_*, 1.0*L_s_*, and 1.2*L_s_*. Among them, 0.8*L_s_* is used to emulate the equivalent inductance reduction caused by magnetic saturation, while 1.2*L_s_* represents parameter deviation on the opposite side.

**Figure 19:**
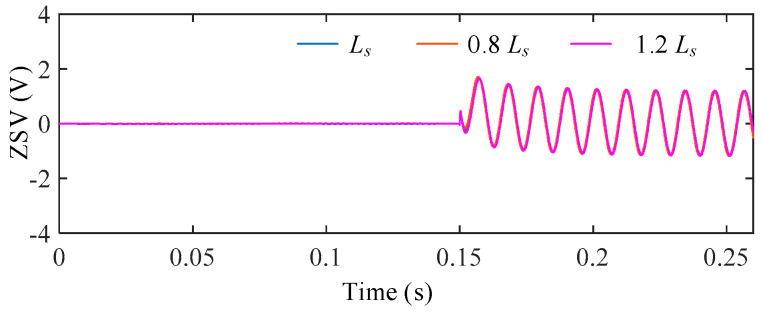
ZSV under different *L_s_*.

**Figure 20:**
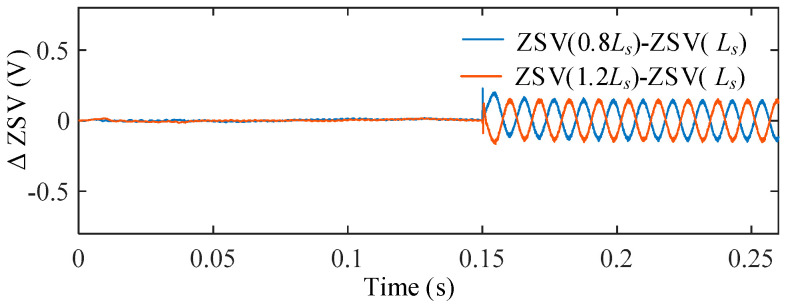
ΔZSV with respect to the nominal *L_s_* under stator inductance variations.

The ZSV under different inductance conditions are shown in Figure 19, and the corresponding difference curves with respect to the nominal case are presented in Figure 20. Compared with the resistance variation case, inductance uncertainty introduces a more noticeable influence on the ZSV magnitude. The ZSV deviation reaches 10.77% under the inductance reduction condition (Ls = 0.8*L_s_*), which corresponds to the magnetic saturation case.

Nevertheless, the ZSV waveforms remain stable without distortion or divergence, indicating that the proposed ZSV estimation can still maintain satisfactory performance under magnetic saturation and inductance uncertainty.

3. Influence of Fault Resistance *R_f_* Variations

**Figure 21:**
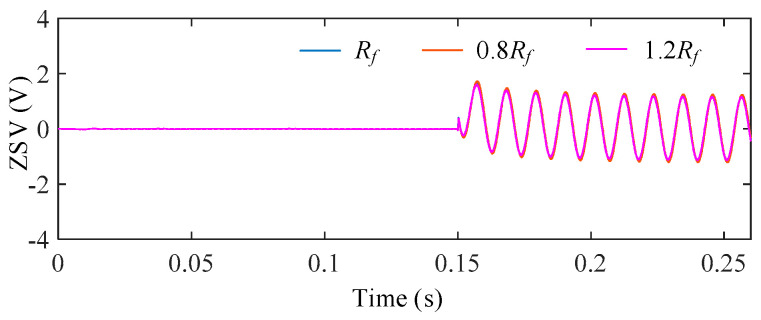
ZSV under different *R_f_*.

**Figure 22:**
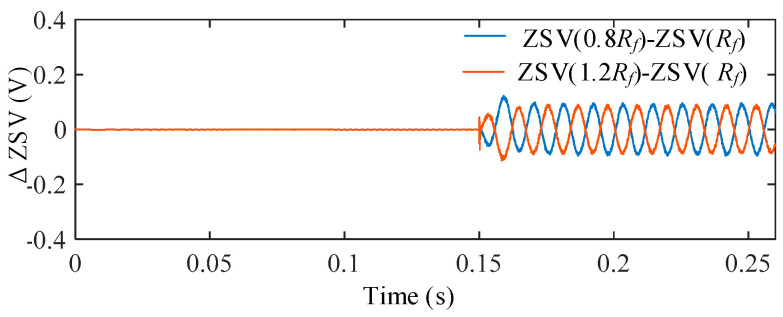
ΔZSV with respect to the *R_f_* under short-circuit resistance variations.

To assess the robustness of the ZSV estimation against the uncertainty of the ITSC fault path, the short-circuit resistance was varied as 0.8*R_f_*, 1.0*R_f_*, and 1.2*R_f_*, representing different short-circuit severities. The resulting ZSV waveforms are depicted in Figure 21, while their deviations from the nominal values are shown in Figure 22.

It can be observed that the ZSV estimation is moderately influenced by the variation of fault resistance. The maximum relative deviation of the ZSV reaches 6.92% compared with the nominal condition. Despite this deviation, the overall trend and stability of the ZSV signal are well preserved, demonstrating adequate robustness against *R_f_* uncertainty.

4. Influence of Inverter Nonlinearities

Inverter nonlinearity is mainly represented by the dead-time effect of power electronic switches. In the simulations, a fixed dead-time of 4 μs was introduced into the PWM signals to emulate a non-ideal inverter operating condition. At the employed switching frequency, this dead-time corresponds to a relatively severe nonlinearity condition. It should be noted that the selected dead-time value is consistent with that used in the experimental setup.

**Figure 23:**
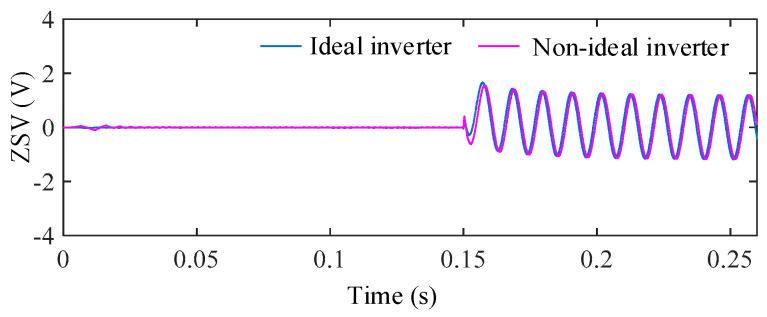
ZSV under ideal inverter operation and non-ideal inverter operation with a dead-time of 4 μs.

**Figure 24:**
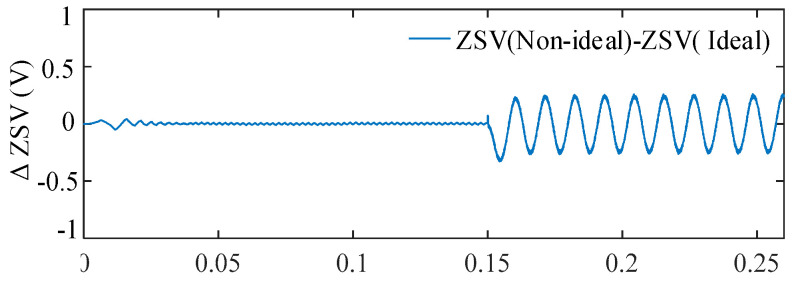
ΔZSV with respect to the ideal inverter under dead-time nonlinearity.

Figure 23 shows the ZSV obtained with and without the dead-time effect. The resultant difference between the two signals is plotted in Figure 24. Among all considered non-ideal factors, inverter dead-time introduces the largest ZSV deviation, with a maximum relative deviation reaching 14.28%. This indicates that inverter nonlinearity is the most influential factor on the ZSV magnitude.

**Figure 25:**
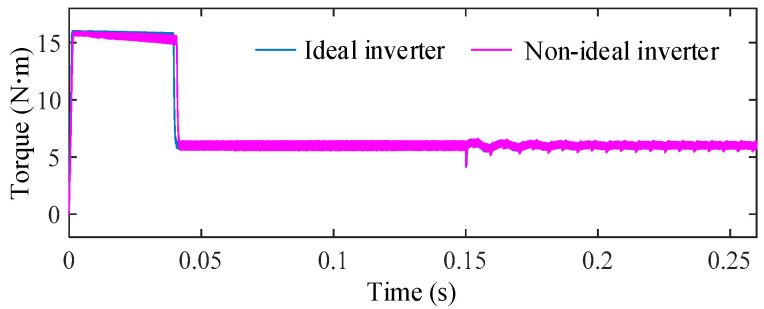
Torque responses under ideal inverter operation and non-ideal inverter operation with a dead-time of 4 μs.

To further evaluate the system-level impact of this worst case ZSV deviation, the torque responses under ideal inverter operation and non-ideal inverter operation with 4 μs dead-time are compared in Figure 25.

It is clearly observed that, despite the significant ZSV deviation, the torque remains smooth and stable under the proposed FTC scheme, exhibiting a torque ripple as low as 3.56%. The absence of any noticeable deterioration confirms the strong robustness of the proposed method against inverter nonlinearities

5. Influence of Measurement Noise

**Figure 26:**
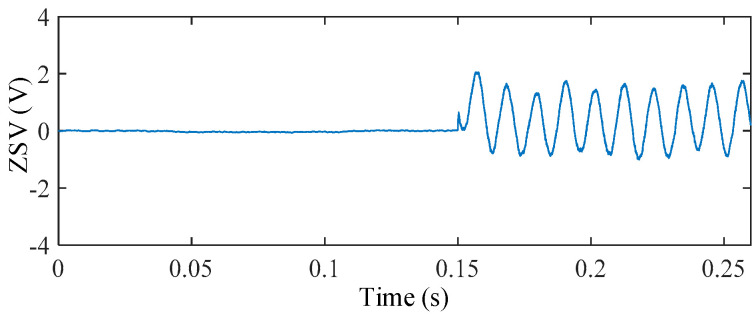
ZSV under measurement noise with an SNR of 30 dB.

To investigate the noise tolerance of the ZSV estimation, band-limited white Gaussian noise was added to the ZSV-based signal, and the signal-to-noise ratio (SNR) was set to 30 dB, representing a typical practical measurement environment. Figure 26 shows the ZSV under noisy conditions, where visible fluctuations are introduced due to measurement noise.

**Figure 27:**
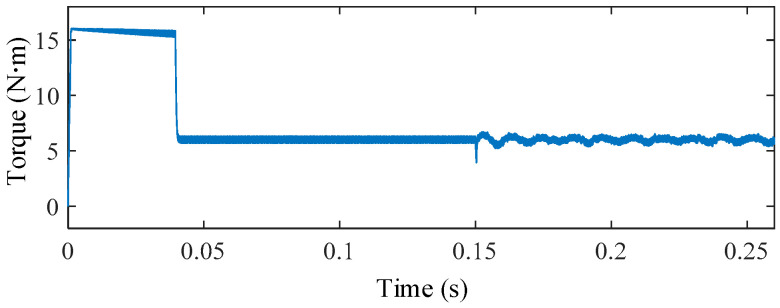
Torque responses under measurement noise with an SNR of 30 dB.

As illustrated in Figure 27, the torque responses under measurement noise confirm that the ripple remains effectively suppressed. A low torque ripple of 3.92% is achieved, indicating strong noise tolerance at the system level for the proposed ZSV estimation and FTC scheme.

Overall, the results further confirm the robustness of the proposed FTC scheme against practical measurement noise.

### 4.2. Experimental Verification

**Figure 28:**
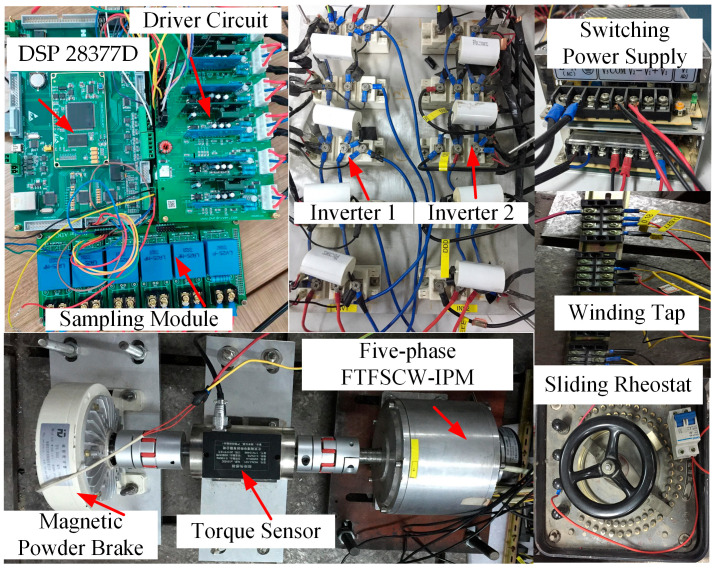
Experimental setup for the OW five-phase FTFSCW-IPM motor drive system.

To further validate the performance of the proposed ZSV-based real-time estimation and FTC method, an experimental platform was established as illustrated in Figure 28.

The proposed control algorithm is implemented on a TMS320F28377D DSP (Texas Instruments, Dallas, TX, USA) operating at 200 MHz and executed in a 10 kHz interrupt cycle. Owing to the low computational complexity of the ZSV estimation and FTC routine, the required computation time corresponds to approximately 10% of the control period, which ensures comfortable real-time implementation for practical motor drive applications.

**Figure 29:**
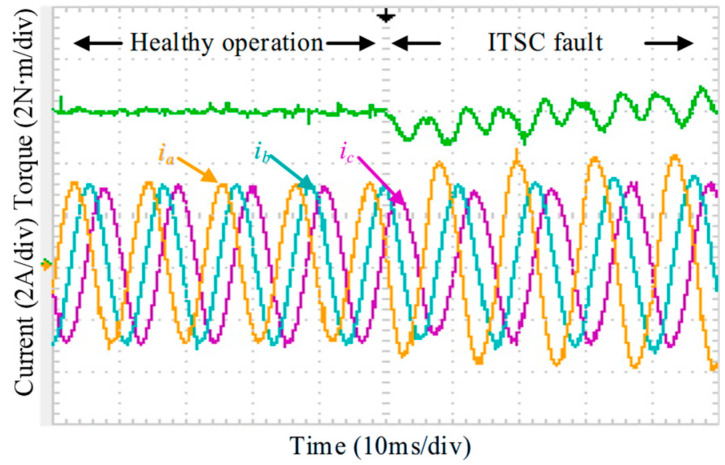
Measured phase currents and torque under the ITSC fault condition.

**Figure 30:**
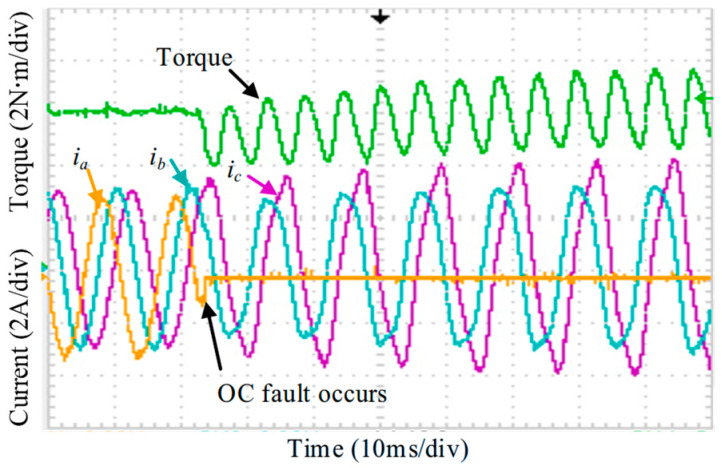
Dynamic phase currents and torque under OC fault.

**Figure 31:**
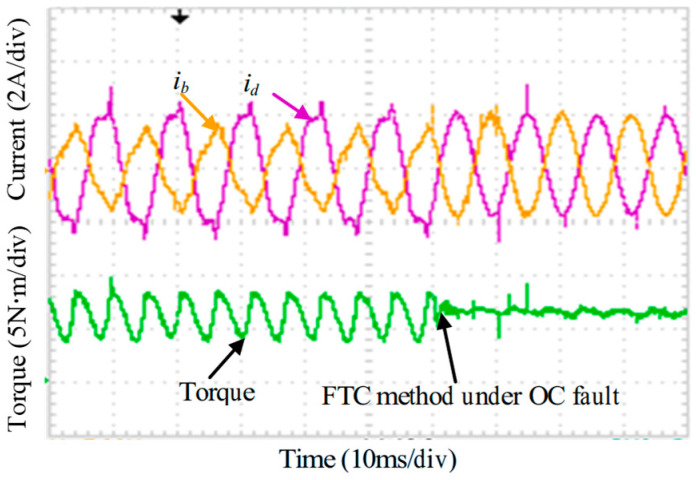
Dynamic phase currents and torque adopt OC FTC method.

**Figure 32:**
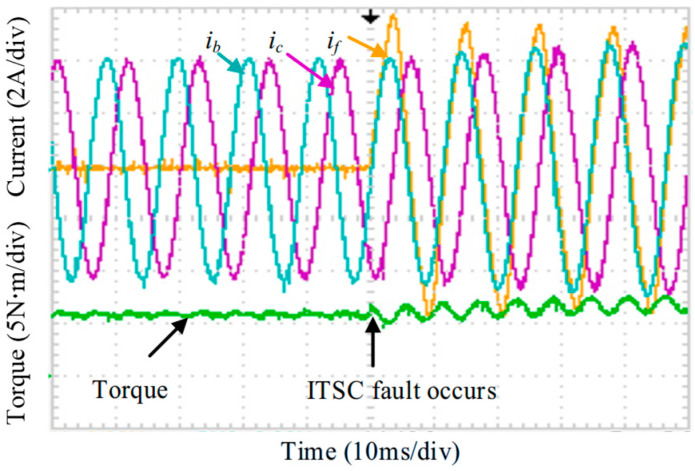
Dynamic currents and torque under ITSC fault.

**Figure 33:**
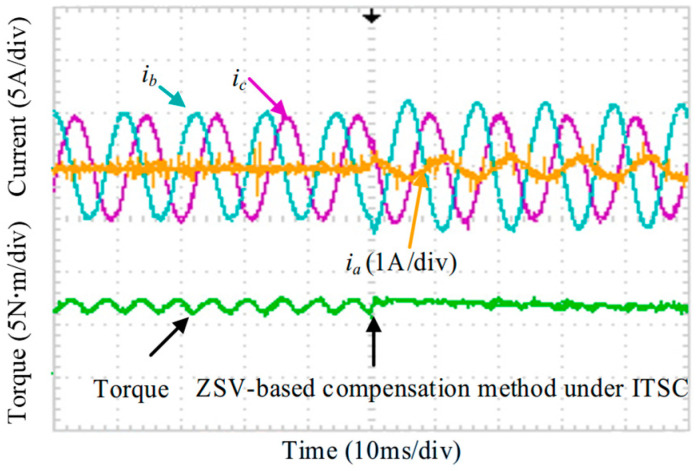
Dynamic currents and torque during compensated FTC operation with real-time estimation incorporated.

**Figure 34:**
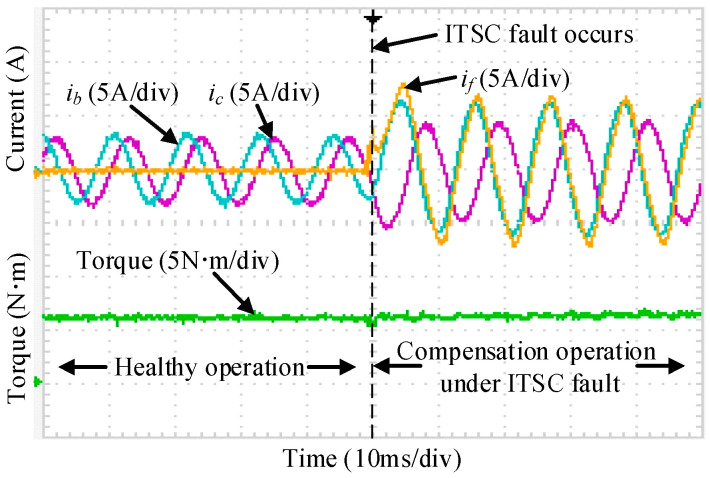
Torque and current response during activation of the ITSC fault-tolerant control.

**Figure 35:**
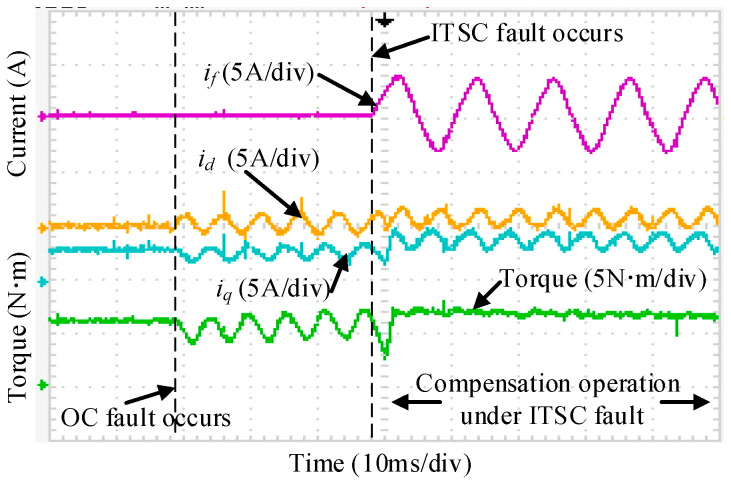
Current and torque response during the transition from healthy operation to OC injection and ITSC fault-tolerant control.

The magnetic powder brake (Pfeiffer Electric Co., Ltd., Wenzhou, China) is used as the load. The sampling module is provided by LEM (LEM International SA, Geneva, Switzerland). Furthermore, a self-built inverter based on IGBT devices from Infineon Technologies (Infineon Technologies AG, Neubiberg, Germany) was used in the experiments. Finally, a sliding rheostat is used as the external resistor following the ITSC fault. Considering the back-EMF magnitude, the value of 150 Ω is selected as a trade-off between limiting the post-fault current for thermal safety and maintaining sufficient ZSV variation for reliable estimation. To emulate different ITSC fault conditions, tap terminals were drawn from various turn positions of the phase-A winding in the five-phase fault-tolerant permanent-magnet motor. The specifications of the motor under test are summarized in Table 1**.** Unless otherwise stated, the experimental conditions for Figure 29, Figure 30, Figure 31, Figure 32, Figure 33, Figure 34 and Figure 35 are: *R_f_* = 2 Ω, *μ* = 0.577, rotational speed *n* = 600 r/min.

Figure 29 shows the measured phase currents and torque during the transition from healthy operation to the onset of the ITSC fault. Once the ITSC fault occurs, the phase currents become severely distorted, and the short-circuit loop causes significant torque disturbance. The resulting torque ripple reaches 38%. Moreover, the current in the faulty phase rises sharply, which may accelerate fault propagation and degrade the drive performance.

In accordance with the analysis in Section 3, the experimental validation is conducted step by step. Figure 30 shows the case of an isolated OC fault without any reconstruction measures. Substantial distortion appears in phases B and C, accompanied by a noticeable torque disturbance, where the torque ripple increases from 3.6% under normal operation to about 34% under the fault condition. When the OC fault FTC strategy is activated, Figure 31 indicates that both the current distortion and the torque disturbance are significantly reduced, with the torque ripple decreased to 4.4%.

With the additional introduction of the ITSC fault, Figure 32 shows the reappearance of the short-circuit loop current in phase A and the resulting renewed torque fluctuation, which elevates the torque ripple to 42%. After connecting phase A to the external resistor and applying the proposed compensation to the d  − q current loop, the results in Figure 33 demonstrate that the torque disturbance is effectively suppressed, resulting in a torque ripple of 5.2%. Due to the external resistor, a small measurable current $i_{A}^{\prime }$ flows through the healthy portion of phase A, which helps mitigate the risk of further insulation degradation and enables the voltage of phase A to be readily calculated.

Since the above tests correspond to sequential activation of individual FTC functions, Figure 34 examines the scenario in which the reconstruction (active OC and external resistor) and the proposed ZSV-based compensation are applied simultaneously under ITSC fault conditions. As shown in Figure 34, the torque remains stable throughout the transition, confirming that the FTC process can be executed smoothly. Because of the injected compensation, the amplitudes of phases B and C differ slightly.

Figure 35 shows the response of the short-circuit current, the d  − q axis currents, and the torque as the system transitions from healthy operation to an injected open-circuit fault and subsequently to ITSC fault-tolerant operation. When phase A is actively opened, noticeable fluctuations appear in the d  − q axis currents, thereby inducing pronounced torque oscillations, and the corresponding torque ripple reaches 36%. After the compensation strategy for the ITSC fault is applied, the torque disturbance is effectively suppressed, and a torque ripple of 4.8% is observed, while a clear compensation component can be identified in the d − q axis currents.

**Figure 36:**
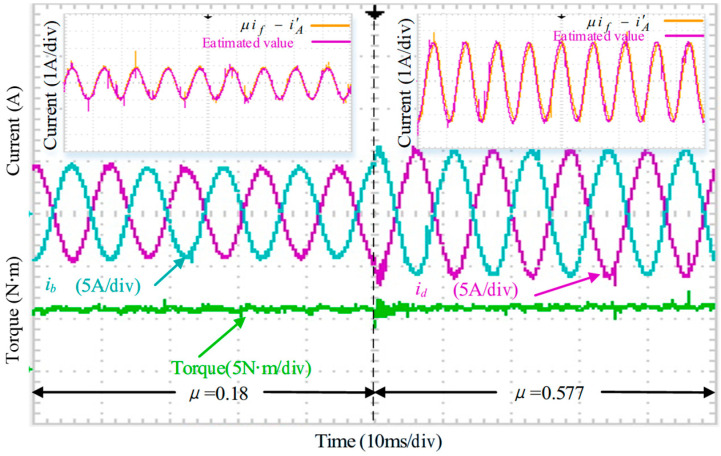
Tracking performance of the feedforward compensation term under varying short-circuit turn ratios.

Figure 36 evaluates the performance of the system under varying short-circuit turn ratios. When *μ* changes from 0.18 to 0.577, the torque remains stable before and after the transition, indicating that the proposed FTC method is effective across different ITSC severity levels.

In addition, the comparison between the estimated term ‘*μi_f_* − $i_{A}^{\prime }$’ and its true value for both *μ* = 0.18 and *μ* = 0.577 shows close agreement, exhibiting behavior consistent with the simulation results in Figure 16.

## 5. Conclusions

This paper presented a fault-tolerant control method for an OW five-phase FTFSCW-IPM motor under ITSC fault conditions. Once the fault is detected, the affected phase is actively opened and connected to an external resistor, enabling both the reduction in the fault-phase current and accurate real-time estimation of the disturbance term through ZSV. A key contribution of this work is provided by the integration of OC fault reconstruction and ZSV-based disturbance estimation into a unified feedforward compensation framework, by which the ITSC induced disturbance is directly compensated without relying on additional voltage sensors or complex observers. As a result, stable post-fault operation is maintained with suppressed torque ripple. Both simulation and experimental results demonstrate the effectiveness and robustness of the proposed method under the considered operating conditions. Moreover, the successful DSP-based implementation verifies its real-time feasibility for practical fault-tolerant motor drive systems.

## Figures and Tables

**Table 1:** Parameters of the five-phase OW FTFSCW-IPM.

Parameter	Value
Stator resistance Rs	0.5 Ω
Self-inductance Ls	13.5 m H
External resistance R	150 Ω/150 W
Mutual inductance Lm	0.035 m H
Rotational inertia J	0.006 kg·m^2^
Flux linkage of permanent magnet	0.089 Wb
Number of pole pairs p	9

## Data Availability

The original contributions presented in this study are included in the article. Further inquiries can be directed to the corresponding author.

## Appendix A. Derivation of the Matrix-Form Control Law

This appendix presents the detailed derivation of the matrix-form control law given in (20). Starting from the individual current error dynamics and PI control laws in the d–q–0 reference frame, the equations are gradually rearranged into a compact vector–matrix form.

When only the OC fault is considered, the Clarke transformation matrix is obtained according to the fault-tolerant control strategy as

(A1) $$T_{F}=\left[\begin{array}{cccc} 0.2236 & -0.2236 & -0.2236 & 0.2236\\ 0.3078 & 0.3078 & -0.3078 & -0.3078\\ 0.1809 & -0.1809 & 0.1809 & -0.1809\\ 0.1809 & 0.1809 & 0.1809 & 0.1809 \end{array}\right]$$

Accordingly, the inverse Clarke transformation matrix is given by

(A2) $$T_{F}^{-1}=\left[\begin{array}{cccc} 1.118 & 0.8123 & 1.382 & 1.382\\ -1.118 & 0.8123 & -1.382 & 1.382\\ -1.118 & -0.8123 & 1.382 & 1.382\\ 1.118 & -0.8123 & -1.382 & 1.382 \end{array}\right]$$

Furthermore, the inverse Park transformation matrix in the d–q–γ–0 subspace is derived as

(A3) $$T_{P}^{-1}=\left[\begin{array}{cccc} \cos\theta & -\sin\theta & 0 & 0\\ \sin\theta & \cos\theta & 0 & 0\\ 0 & 0 & 1 & 0\\ 0 & 0 & 0 & 1 \end{array}\right]$$

Since a PI controller is adopted in the post-fault current regulation, the reference voltage in the d–q–γ–0 plane is expressed as

(A4) $$\begin{array}{l} \left[\begin{array}{c} u_{dref}\\ u_{qref}\\ u_{\gamma ref}\\ u_{0ref} \end{array}\right]=\left[\begin{array}{c} \left(k_{dp}+\frac{k_{di}}{s})\times (i_{d}^{*}-i_{d}\right)\\ \left(k_{qp}+\frac{k_{qi}}{s})\times (i_{q}^{*}-i_{q}\right)\\ k_{\gamma p}+\frac{k_{\gamma i}}{s}\\ k_{op}+\frac{k_{0i}}{s} \end{array}\right] \end{array}$$

After the ITSC fault occurs, by taking into account the feedforward compensation effect, the reference voltage is modified as

(A5) $$\begin{array}{l} \left[\begin{array}{c} u_{dref}\\ u_{qref}\\ u_{\gamma ref}\\ u_{0ref} \end{array}\right]=\left[\begin{array}{c} \left(k_{dp}+\frac{k_{di}}{s})\times [i_{d}^{*}-i_{d}+0.4(\mu i_{f}-{i^{\prime }}_{A})\cos\theta \right]\\ \left(k_{qp}+\frac{k_{qi}}{s})\times [i_{q}^{*}-i_{q}-0.4(\mu i_{f}-{i^{\prime }}_{A})\sin\theta \right]\\ k_{\gamma p}+\frac{k_{\gamma i}}{s}\\ k_{op}+\frac{k_{0i}}{s} \end{array}\right] \end{array}$$

The reference voltage in the d–q–γ–0 plane is then transformed into the stationary reference frame through the transformation matrices (A2) and (A3). By further incorporating the back electromotive forces, the reference voltage in the stationary coordinate system is obtained as

(A6) $$\begin{array}{l} \left[\begin{array}{c} u_{B}^{*}\\ u_{C}^{*}\\ u_{D}^{*}\\ u_{E}^{*} \end{array}\right]=T_{F}^{-1}T_{P}^{-1}\left[\begin{array}{c} u_{dref}\\ u_{qref}\\ u_{\gamma ref}\\ u_{0ref} \end{array}\right]+\left[\begin{array}{c} e_{B}\\ e_{C}\\ e_{D}\\ e_{E} \end{array}\right] \end{array}$$

Finally, by substituting (A5) into (A6), the final voltage expression is derived as

(A7) $$\begin{array}{l} \left[\begin{array}{c} u_{B}^{*}\\ u_{C}^{*}\\ u_{D}^{*}\\ u_{E}^{*} \end{array}\right]=T_{F}^{-1}T_{P}^{-1}\left[\begin{array}{c} \left(k_{dp}+\frac{k_{di}}{s})\times [i_{d}^{*}-i_{d}+0.4(\mu i_{f}-{i^{\prime }}_{A})\cos\theta \right]\\ \left(k_{qp}+\frac{k_{qi}}{s})\times [i_{q}^{*}-i_{q}-0.4(\mu i_{f}-{i^{\prime }}_{A})\sin\theta \right]\\ k_{\gamma p}+\frac{k_{\gamma i}}{s}\\ k_{op}+\frac{k_{0i}}{s} \end{array}\right]+\left[\begin{array}{c} e_{B}\\ e_{C}\\ e_{D}\\ e_{E} \end{array}\right] \end{array}$$

It can be observed that the obtained matrix-form voltage expression in (A7) corresponds to the compact control law given in (20) in the main text, thereby completing the detailed derivation process.
